# Inter-observer variability of clinical target volume delineation in radiotherapy treatment of pancreatic cancer: a multi-institutional contouring experience

**DOI:** 10.1186/1748-717X-9-198

**Published:** 2014-09-08

**Authors:** Luciana Caravatta, Gabriella Macchia, Gian Carlo Mattiucci, Aldo Sainato, Nunzia LV Cernusco, Giovanna Mantello, Monica Di Tommaso, Marianna Trignani, Antonino De Paoli, Gianni Boz, Maria L Friso, Vincenzo Fusco, Marta Di Nicola, Alessio G Morganti, Domenico Genovesi

**Affiliations:** Radiation Oncology Department, “San Francesco” Hospital, Via Mannironi, 1, 08110 Nuoro, Italy; Radiation Oncology Department, Fondazione di Ricerca e Cura “Giovanni Paolo II”, Università Cattolica del S. Cuore, Campobasso, Italy; Radiotherapy Department, Università Cattolica del S. Cuore, Roma, Italy; Radiotherapy Unit, Azienda Ospedaliera Universitaria Pisana, Pisa, Italy; Department of Radiotherapy, State Hospital, Ancona, Italy; Department of Radiotherapy, “SS Annunziata” Hospital, “G. D’Annunzio” University, Chieti, Italy; Department of Radiation Oncology Centro di Riferimento Oncologico, National Cancer Institute, Aviano, Italy; Radiotherapy and Nuclear Medicine Unit, Istituto Oncologico Veneto, IRCCS, Padova, Italy; Department of Radiation Oncology, IRCCS CROB, Rionero in Vulture, Potenza, Italy; Department of Experimental and Clinical Sciences, Laboratory of Biostatistics, “G. D’Annunzio” University, Chieti, Italy

## Abstract

**Background:**

An observational multi-institutional study has been conducted aimed to evaluate the inter-observer variability in clinical target volume (CTV) delineation among different radiation oncologists in radiotherapy treatment of pancreatic cancer.

**Methods:**

A multi-institutional contouring dummy-run of two different cases of pancreatic cancer treated by postoperative and preoperative radiotherapy (RT) was performed. Clinical history, diagnostics, and planning CT imaging were available on AIRO website (http://www.radioterapiaitalia.it). Participants were requested to delineate CTVs according to their skills and knowledge. Aiming to quantify interobserver variability of CTVs delineations, the total volume, craniocaudal, laterolateral, and anteroposterior diameters were calculated. Descriptive statistic was calculated. The 95% Confidence Interval (95% CI) for coefficient of variation (CV) was estimated. The Dice Similarity Index (DSI) was used to evaluate the spatial overlap accuracy of the different CTVs compared with the CTVs of a national reference Centre considered as a benchmark. The mean DSI (mDSI) was calculated and reported.

**Results:**

A total of 18 radiation oncologists from different Institutes submitted the targets. Less variability was observed for the Elective CTV rather than the Boost CTV, in both cases. The estimated CV were 28.8% (95% CI: 21.2 - 45.0%) and 20.0% (95% CI: 14.9 - 30.6%) for the Elective CTV, in adjuvant (Case 1) and neoadjuvant (Case 2) case, respectively. The mDSI value was 0.68 for the Elective CTVs in both cases (range 0.19 - 0.79 in postoperative vs range 0.35 - 0.79 in preoperative case). The mDSI was increased to 0.71 (Case 1) and 0.72 (Case 2) if the observers with a worse agreement have been excluded. On the other hand, a CV of 42.4% (95% CI: 30.1 - 72.4%) and 63.8% (95% CI: 43.9 - 119.2%) with a mDSI value of 0.44 and 0.52, were calculated for the Boost CTV in Case 1 and Case 2, respectively.

**Conclusions:**

The CV and mDSI obtained values for Elective CTVs showed an acceptable agreement among participants either in postoperative as well in preoperative setting. Additional strategies to reduce the variability in Boost CTV delineation need to be found and promoted.

## Background

Patients with pancreatic adenocarcinoma (PC) have a poor prognosis. In an attempt to improve survival, chemotherapy and radiotherapy (RT) have been used both for unresectable disease as well as in the adjuvant setting [1, 2]. About one third of PC patients die from local uncontrolled disease and lymph node metastases have been proved to be an important prognostic factor associated with a significantly higher rate of both local and distant recurrences [3]. Therefore, local control remains an important treatment end-point.

Although regional nodal metastases are often found in patients with PC, it remains debatable whether elective nodal irradiation (ENI) should be performed. Since the high reported frequency of lymphatic spread (60–80%) in head pancreatic cancer [4] and the high rate of local and nodal failure reported in pathologic and clinical analyses (up to 75%) [5], elective ENI seems to be justified in a curative treatment.

However, one of the major RT challenges for the upper abdominal tumor, especially if ENI is required, is the radio-sensibility of multiple critical structures, including liver, kidneys, stomach, small bowel, and spinal cord. Intensity-modulated radiation therapy (IMRT) has been shown to reduce dose to organs at risk (OARs) [6–8] improving planning target volume (PTV) coverage [9].

Due to the deep dose gradients between the boundary of target volumes and OARs, more than those obtained by conventional RT, a higher accuracy in the delineation of the clinical target volume (CTV) become a fundamental prerequisite [10]. In the last decade, Brunner and colleagues proposed guidelines for definition of ENI target volume in PC [11]. More recently, Radiation Therapy Oncology Group consensus panel guidelines for the delineation of the CTV in the postoperative treatment of pancreatic head cancer were published [12]. In addition, Sun et al. reviewed 18 pathological reports accounting for 5954 PC patients treated with radical surgery. The probability of metastasis in regional lymph nodal stations (using Japan Pancreas Society [JPS] Classification) was calculated and analyzed based on the location and other characteristics of the primary disease. Site and probability of metastasis were identified and suggested as a guide for surgical treatment [13]. Based on this review, Caravatta et al. proposed an atlas reporting criteria for CTV, including ENI definition and delineation in the preoperative or exclusive treatment of PC [14].

In 2012, the Gastrointestinal Study Group of the Italian Association of Radiation Oncology (AIRO-GI) promoted the recently published guidelines [12, 14] to National RT centres as contouring tools for RT in PC [15]. After 1 year, in order to highlight the CTV delineation uncertainties among different radiation oncologists of the available contouring guidelines, a multi-institutional contouring dummy-run of 2 different PC cases treated by preoperative and postoperative RT was proposed.

## Material and method

### Qualitative analysis

A structured questionnaire was administered. The professional seniority, the number of per year-treated patients, some technical details (e.g. the use of intravenous contrast-enhanced planning CT scans and of multi modality imaging), the existence of multidisciplinary team for contouring, were investigated. Noteworthy, a detailed definition of the anatomical sites encompassed in the CTVs in daily clinical practice was demanded, as for Gross tumor volume (GTV), as well for tumor bed and lymph node areas, according to JPS nomenclature [16].

### Contouring section

A national reference RT centre was identified on the basis of per-year PC treated patients (more than 30) and of PC expertise according to scientific publications on the topic. Contouring data from the latter were considered as the benchmark in the comparison analysis.

### Clinical cases

Two PC cases were chosen in postoperative and in preoperative setting. Data on clinical history, staging, imaging (CT, MRI) and planning CT were available for participants on AIRO website (http://www.radioterapiaitalia.it). Participant’s centres were requested to delineate target volumes by their own segmenting tools and clinical experience based on specific instructions in terms of guidelines or recommendations [15]. The planning CT scan was obtained with a standard acquisition protocol (supine position with the arms elevated; slice thickness of 5 mm and reconstruction interval of 5 mm). Oral or intravenous contrast mediums were not administered.

### Case 1

In July 2012, a 69-year-old woman was hospitalized for fatigue, progressive jaundice, pale stools, dark urine and itch. Abdomen CT scan showed into the head and uncinate process of the pancreas an expansive solid lesion (maximum diameter = 27 mm) associated with dilatation of the intrahepatic bile ducts. Some small lymph nodes were detectable around the hepatoduodenal ligament, celiac trunk, inter-aorto-caval and paraortic areas. At the endoscopic ultrasound procedures a lymph node (maximum diameter = 8.5 mm) with doubtful characteristics of malignancy was also identified close to duodenum. Since the diagnosis of pancreatic adenocarcinoma was confirmed by fine-needle aspiration (FNA) biopsy, the patient underwent to pancreaticoduodenectomy. The pathology reports documented a ductal pancreatic adenocarcinoma, G2, with perineural invasion, extensively infiltrating the parenchyma and extended to the subserosal and retropancreatic adipose tissue up to the muscle layer of the duodenal wall. Surgical margins were negative. Lymph node metastases were recognized in two of four resection specimens of retropancreatic area, while 8 additional nodes were metastasis-free. Stage according to the 7^th^ edition of the AJCC TNM staging system was pT3, pN1. Participants were required to encompass two CTVs: 1) high risk draining lymph nodes areas (Elective CTV); 2) tumor bed plus posterior pancreaticoduodenal lymph nodes (Boost CTV).

### Case 2

In March 2012, a 72-year-old man was hospitalized for obstructive jaundice. Abdomen CT scan showed a 3.0 × 25 × 35 mm hypodense solid lesion, at the uncinate process of the pancreas, with the invasion of confluence of superior mesenteric and portal vein. At the endoscopic ultrasound procedure 2 lymphadenopathies with characteristics of malignancy were described in the intercavoaortic space and in the hepatic hilum. Patient was staged as cT3, cN1, according to the 7^th^ edition of AJCC TNM staging system. The diagnosis of PC adenocarcinoma was confirmed by FNA biopsy. Participants were required to encompass two target volumes: 1) high risk draining lymph nodes areas (Elective CTV); 2) detectable tumor plus positive lymph nodes (Boost CTV).

### Metodology

Contoured CTVs from partecipant’s centers were loaded on Oncentra® Masterplan Treatment Planning System (Nucletron) for geometrical parameters analysis (total volume, craniocaudal, laterolateral, and anteroposterior diameters) and on Eclipse® TPS (Varian) to calculate the mean DSI’s Similarity Index (mDSI).

As in some our previous experiences [17, 18] differences in CTVs cranio-caudal extension (i.e., the number of slices contoured multiplied by the slice thickness), maximum latero-lateral diameter, and maximum anterio-posterior diameter were calculated. Moreover, a comparison between each cranial and caudal limits of the CTVs, maximum anterio-posterior diameter (extended from the extreme anterior point to the extreme posterior point of the CTVs), and maximum latero-lateral diameter (extended from the extreme point on the right to the extreme point on the left of the CTVs) were evaluated. Descriptive statistics (minimum, maximum, mean, standard deviation, median, 25^th^ and 75^th^ percentile and coefficient of variation [CV]) was calculated for each parameter. Scatter plots were used for the presentation of each CTV showing median values and the 25^th^ and 75^th^ percentile range. A Shapiro-Wilk’s test was performed to evaluate the deviation from normality distribution for each parameter. The 95% Confidence Interval (95% CI) for CV and for individual predicted volume were estimated using non central-t distribution and left-truncated normal distribution (where the fixed-point of truncation was zero), respectively. The comparison between relative variations was evaluated using the Student t distribution. Statistical analysis was performed using SPSS® Advanced Statistical 11.0 software (SPSS Inc, Chicago, Illinois, USA) and R open source software.

### Dice Similarity Index (DSI)

The DSI was used as a statistical validation metric to evaluate the spatial overlap accuracy of the different delineations of CTVs [19, 20] and compared with the contouring of the reference centre considered as the benchmark. Given two observers contouring the volumes A and B, DSI is defined as:


The value of a DSI is a scalar coefficient ranges from 0, indicating no spatial overlap between two sets of binary segmentation results, to 1, indicating complete overlap.

## Results

Without any predetermined selection criterion, 18 radiation oncologists from different centres spontaneously submitted the completed questionnaire and the delineated targets.

Qualitative data from participating centers have been detailed in Table 1. Senior doctors with experience on PC longer more than 10 years were 44.4%. All respondents declared to use the staging imaging (CT scan) for the delineation of CTVs in both setting. In particular, 8 centres (44.4%) declared to never or rarely require multi-modality imaging (RM or CT-PET) for CTVs delineation in routine clinical practice, whereas multi-modality imaging was considered necessary for selected cases in 7 centres (38.9%). Co-registration with the planning CT scan resulted routinely performed in 6 centres (33.3%). Fifteen radiation oncologists (83.3%) stated that the collaboration with radiologists and/or nuclear physician for CTVs delineation was required only for very difficult interpretation cases.

**Table 1:** **Qualitative analysis results**

Questionnaire		Professional seniority				N° tratment/year				IV contrast-enhanced planning CT scan			Multi-Modality Imaging (MRI or CT-PET)			
		Senior resident	< 5 years specialist	5-10 years specialist	>10 years specialist	<5	5-10	10-30	>30	Never	Selected cases	Always	Never	Almost Never	Selected cases	Always
n	18	3	7	0	8	7	5	4	2	7	5	6	5	3	7	3
%	100	16.7	38.9	0	44.4	38.9	27.8	22.2	11.1	38.9	27.8	33.3	27.8	16.7	38.9	16.7

IV: intravenous.

Concerning the definition of the Elective CTV, 15 radiation oncologists stated to include 6, 8, 9 ,12, 13, 14 16 and 17 lymph nodes groups for head pancreatic cancer and 6, 8, 9 ,10, 11, 12, 14 16 and 18 lymph nodes groups (JPS classification) for body/tail pancreatic cancer, in both setting. Three radiation oncologists declared to avoid contouring of infrapyloric lymph nodes (group 6) in both treatment setting, irrespective of primary tumor site.

### Case 1: post-operative RT

**Figure 1:**
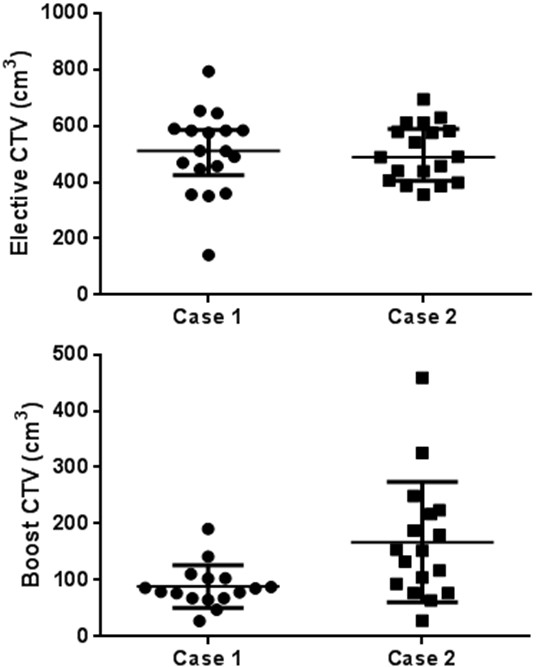
**Scatter plots of CTVs for Elective and Boost CTVs of Case 1 and 2 delineated by each observer.**

**Figure 2:**
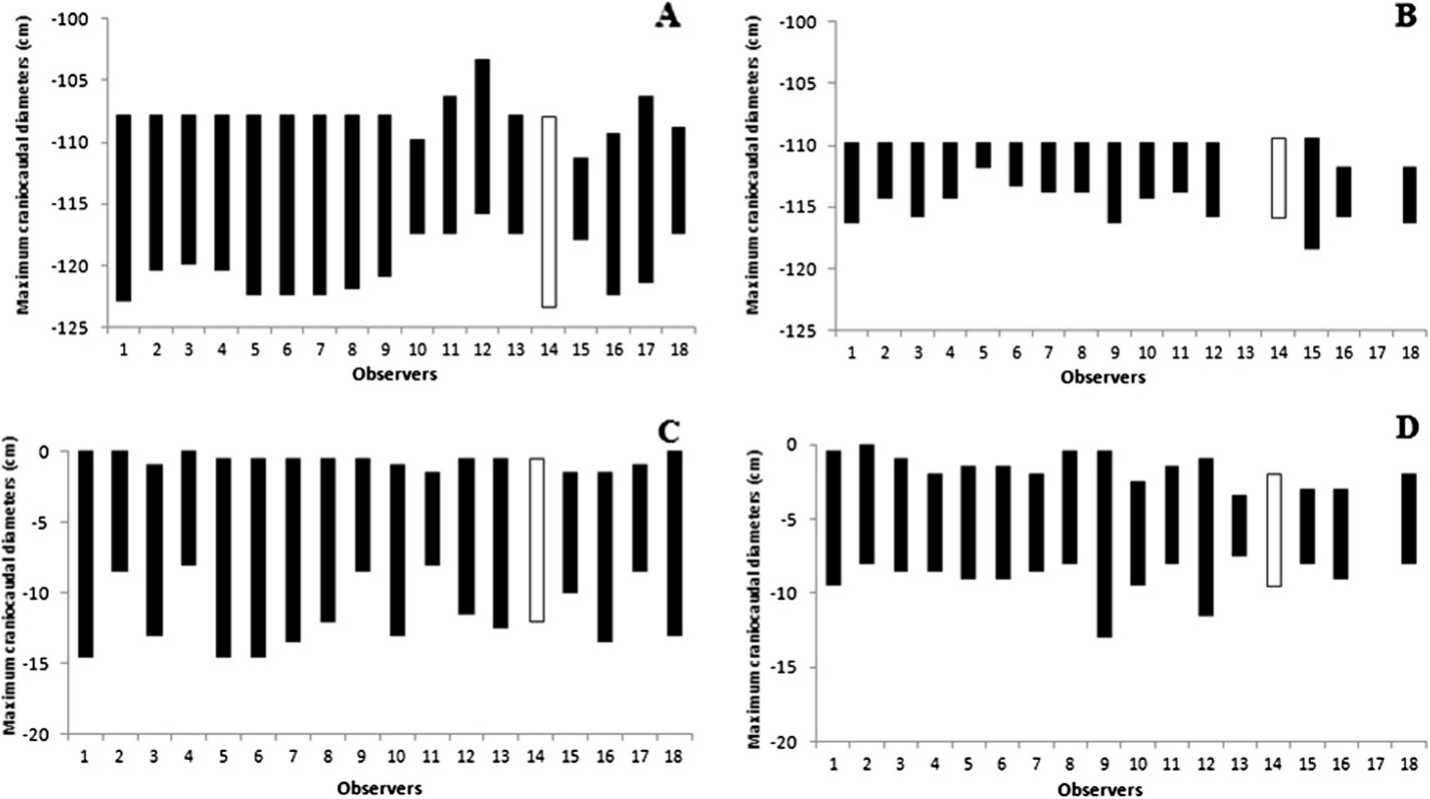
**Maximum craniocaudal diameters for Elective and Boost CTVs of Case 1** **(Panel A and B, respectively)** **and 2** (**Panel C and D,** **respectively)** **delineated by each observer.** The center of reference is represented in white.

**Figure 3:**
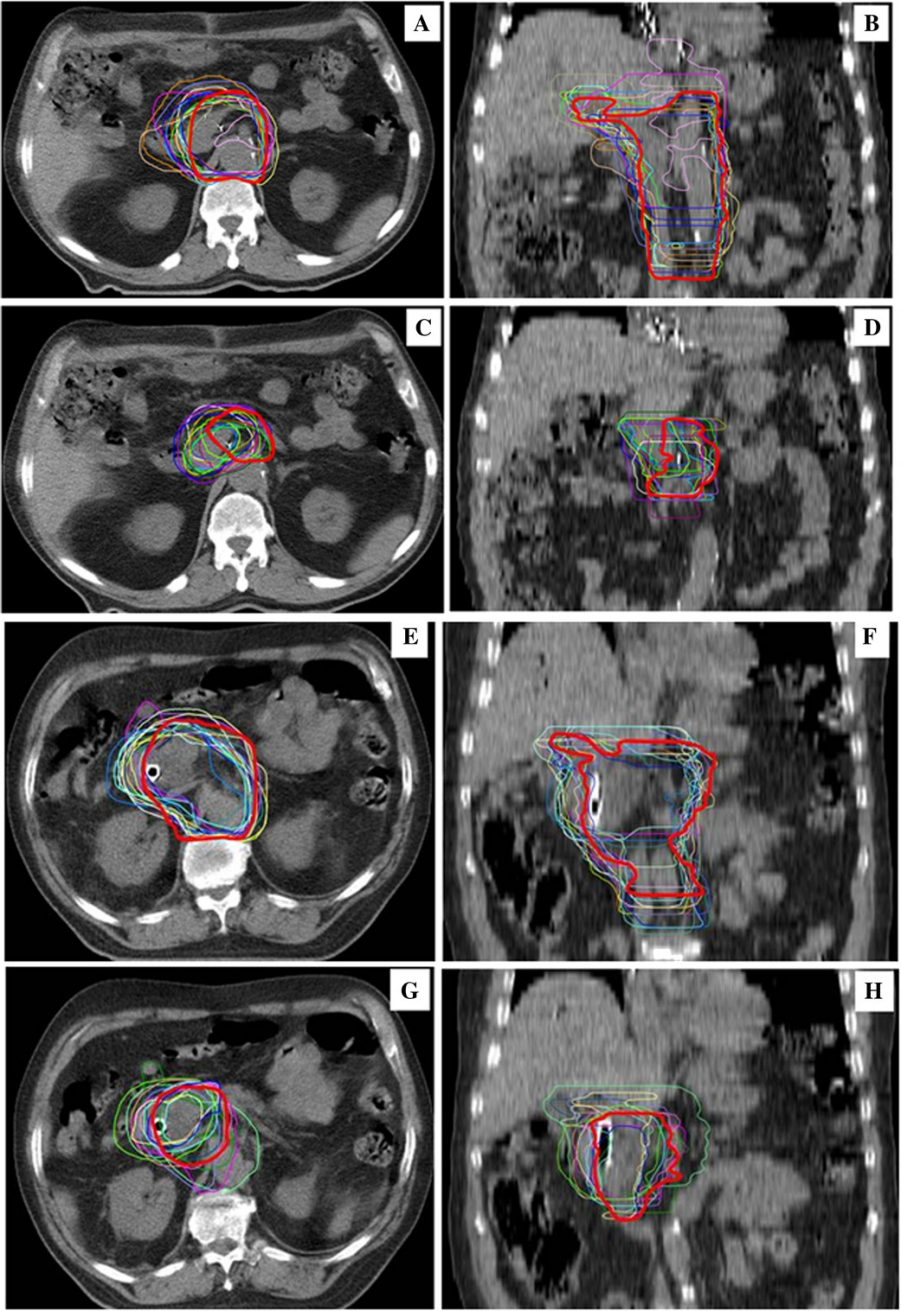
**Graphic representation on axial (Panel A, C, E and G) and coronal (Panel B, D, F and H) planes of interobserver variation between 18 centres for Elective** **(Panel A and B)** **and Boost** **(Panel C and D)** **CTVs of post**-**operative radiotherapy (Case 1) and for Elective (Panel E and F) and Boost** **(Panel G and H)** **CTVs of pre-operative radiotherapy (Case 2).** The center of reference is represented in red solid outline.

Two of the 18 centres did not delineate the Boost CTV. The mean Elective CTV and Boost CTV volumes were 505.4 cm^3^ and 88.8 cm^3^, with a standard deviation (SD) of 145.7 cm^3^ (range 141.8 - 792.7 cm^3^) and of 37.7 cm^3^ (range 27.9 - 191.1 cm^3^), respectively (Table 2). Particularly 4 of 18 centres delineated an Elective CTV with a volume ≤424.5 cm^3^ (25^th^ percentile) and 4 of 18 centres with a volume ≥585.3 cm^3^ (75^th^ percentile), whereas 3 of 16 centres delineated a Boost CTV ≤68.0 cm^3^ (25^th^ percentile) and 4 of 16 centres with a volume ≥103.3 cm^3^ (75^th^ percentile) (Table 2 and Figure 1). The 95% CI for each individual predicted volume were 432.9 - 577.9 cm^3^ for the Elective CTV and 68.7 - 108.9 cm^3^ for the Boost CTV. The estimated CV% were 28.8% (95% CI: 21.2 - 45.0%) for the Elective CTV and 42.4% (95% CI: 30.1 - 72.4%) for the Boost CTV, respectively. No significant difference was detected between the CVs. The variation of both CTVs concerning cranio-caudal, anterio-posterior, and latero-lateral diameters is also shown in Table 2. Moreover, the variability of cranial and caudal limits drawn by each observer on CT slices is represented in Figure 2, showing as the greatest variability was in the caudal direction, especially for Boost CTV. Deviation from Elective CTV and Boost CTV volumes, evaluated by the reference centre, were −83.1 ± 148.8 (range: −442.1 to 208.8) and 0.8 ± 39.0 (range: −60.2 to 103.0), respectively. In both cases, 5 physicians have delineated a larger volume than the reference centre. A graphic representation on axial and coronal planes of interobserver variation among 18 centres for Case 1 Elective and Boost CTVs is shown in Figure 3.

**Table 2:** **Volumetric and dimensional results for clinical target volumes** (**CTVs**) **of Case 1 and 2**

Case 1	Elective CTV				Boost CTV			
	Volume (cm ^3^)	Laterolateral diameter (cm)	Anteroposterior diameter (cm)	Craniocaudal diameter (cm)	Volume (cm ^3^)	Laterolateral diameter (cm)	Anteroposterior diameter (cm)	Craniocaudal diameter (cm)
N	18	18	18	18	16	16	16	16
Mean	505.4	14.1	10.2	12.4	88.8	7.2	6.8	5.1
SD	145.7	2.0	1.9	2.7	37.7	1.1	1.7	1.6
Minimum	141.8	11.9	5.4	6.5	27.9	4.8	5.3	2.0
Maximum	792.7	19.1	15.0	15.5	191.1	9.0	11.9	9.0
25^th^ percentile	424.5	12.3	9.3	10.6	68.0	6.5	5.8	4.0
Median	510.8	13.6	10.3	12.7	82.4	7.3	6.3	4.5
75^th^ percentile	585.3	15.3	11.0	15.0	103.3	8.2	6.9	6.0
CV (%)	28.8	14.2	18.6	21.8	42.4	15.3	25.0	31.4
**Case 2**	**Elective CTV**				**Boost CTV**			
	**Volume (cm** ^**3**^ **)**	**Laterolateral diameter (cm)**	**Anteroposterior diameter (cm)**	**Craniocaudal diameter (cm)**	**Volume (cm** ^**3**^ **)**	**Laterolateral diameter (cm)**	**Anteroposterior diameter (cm)**	**Craniocaudal diameter (cm)**
N	18	18	18	18	17	17	17	17
Mean	503.9	14.2	11.7	11.0	167.4	8.6	7.4	7.3
SD	101.0	2.3	1.8	2.5	106.9	2.3	1.6	2.0
Minimum	356.0	10.9	8.7	6.5	28.3	4.1	4.5	4.0
Maximum	693.6	18.2	15.6	14.5	458.9	13.0	9.9	12.5
25^th^ percentile	404.3	12.4	10.6	8.4	85.5	6.8	6.4	6.2
Median	489.0	14.0	11.2	11.7	152.2	9.0	7.2	7.5
75^th^ percentile	588.7	16.7	12.3	13.0	220.6	10.0	9.1	7.7
CV (%)	20.0	16.2	15.4	22.7	63.8	26.7	21.6	27.4

The DSI was obtained for each centre comparing all CTVs drawn by each observer with the CTVs drawn by the reference centre. The mean DSI values of 0.68 (range: 0.19 - 0.79) for the Elective CTV and 0.44 (range: 0.17 - 0.65) for the Boost CTV were respectively calculated (Table 3). A subsequent analysis, performed after exclusion of data from a single centre whose Elective CTV substantially diverged from the reference, showed an improved agreement of the mDSI value from 0.68 to 0.71.

**Table 3:** **DiceSimilarity Index** (**DSI**) **values for Elective and Boost CTV for Case 1 and Case 2**

Centre	Elective CTV		Boost CTV	
	Case 1	Case 2	Case 1	Case 2
1	0.66	0.68	0.63	0.52
2	0.74	0.6	0.25	0.39
3	0.73	0.79	0.65	0.67
4	0.74	*0.57	0.25	0.56
5	0.70	0.71	0.17	0.51
6	0.74	0.73	0.30	0.61
7	0.75	0.74	0.44	0.76
8	0.76	0.78	0.49	0.53
9	0.74	0.78	0.51	0.63
10	0.69	0.74	0.47	0.61
11	0.63	*0.35	0.41	0.49
12	*0.19	0.62	0.42	0.54
13	0.64	0.68	ND	0.31
14	RC	RC	RC	RC
15	0.65	0.75	0.47	0.68
16	0.79	0.75	0.54	0.63
17	0.70	0.76	ND	ND
18	0.66	0.73	0.54	0.7
*mDSI*	0.68	0.68	0.44	0.52
*range*	(0.19 - 0.79)	(0.35 - 0.79)	(0.17 - 0.65)	(0.31 - 0.76)

mDSI: mean DSI; RC: reference centre; ND: not delineated; centers significantly diverged from the reference (asterisk).

### Case 2: pre-operative RT

One of the 18 centres did not delineate the Boost CTV for the preoperative case. The mean Elective CTV and Boost CTV volumes were 503.9 cm^3^ and 167.4 cm^3^, with a standard deviation (SD) of 101.0 cm^3^ (range 356.0 - 693.6 cm^3^) and of 106.9 cm^3^ (range 28.3 - 458.9 cm^3^), respectively (Table 2). Particularly 4 of 18 centres delineated an Elective CTV with a volume ≤404.3 cm^3^ (25^th^ percentile) and 4 of 18 centres with a volume ≥588.7 cm^3^ (75^th^ percentile), whereas 4 of 17 centres delineated a Boost CTV ≤85.5 cm^3^ (25^th^ percentile) and 4 of 17 centres with a volume ≥220.6 cm^3^ (75^th^ percentile) (Table 2 and Figure 1). The 95% CI for each individual predicted volume were 453.7 - 554.2 cm^3^ for the Elective CTV and 112.4 - 222.3 cm^3^ for the Boost CTV. The estimated CV% were 20.0% (95% CI: 14.9 - 30.6%) for the Elective CTV and 63.8% (95% CI: 43.9 - 119.2%) for the Boost CTV, respectively. A statistically significant difference between the CVs was detected (p < 0.001). The variation of both CTVs concerning cranio-caudal, anterio-posterior, and latero-lateral diameters, were also shown in Table 2. A representation of the variability of cranial and caudal limits drawn by each observer on CT slices is shown in Figure 2. As for post-opertative case, the greatest variability has been shown in the caudal direction, for both volumes. Deviation from volume of Elective CTV and Boost CTV, evaluated by the reference centre, were −39.7 ± 103.7 (range: −185.4 to 152.2) and −16.1 ± 110.3 (range: −123.9 to 306.7), respectively. In both cases, 8 physicians have delineated a larger volume than the reference centre. A graphic representation on axial and coronal planes of interobserver variation between 18 centres for Elective and Boost CTVs of Case 2 is represented in Figure 3.

The mean DSI was calculated for all CTVs drawn by each observer and was 0.68 (range: 0.35 - 0.79) for the Elective CTV and 0.52 (range: 0.31 - 0.76) for the Boost CTV, respectively (Table 3). As for Elective CTV in Case 1, only 2 centres significantly diverged from the reference. The mDSI value excluding these centers was recalculated, resulting increased from 0.68 to 0.72.

## Discussion

Loco-regional recurrence in resected PC is a significant problem with a reported rate of 70–80% [1–5]. Aiming to improve tumor local control with lower toxicities by conformal RT or IMRT technique, an extremely accuracy in definition and delineation of target volumes and risk structures is required. The promotion of contouring guidelines might help to achieve this goal, as well as to reduce the observational variability [11–15] and the impact that different contouring could have on the dose distribution to the CTV and the OAR [21]. Indeed, increasing data showed that the technical quality and administration of radiation therapy or deviations from established QA guidelines had have a relevant impact on clinical outcomes [22] and that standardized atlases of critical radiologic anatomy tailored for radiation therapy and case examples could improve protocol treatment compliance [23].

Until few years ago, the CTV definition in PC has been referred to bone boundaries, so that resulting in conventional large-field radiation treatment [24, 25]. Since a considerable inter-individual anatomical variability for the abdominal vessels was shown with a substantial variability in CTV, RT planning for regional lymphatic of the upper abdomen should be based on identifiable anatomical regions of interest [11, 26]. On the basis of these considerations, some CTV delineation criteria have been selected and proposed as national guideline [12–15].

In our study, we noted a relatively low variability of the inter-observer delineation of the Elective CTVs, in both cases, as expressed by the CV (28.8% and 20.0% in post-operative and pre-operative case, respectively). These results could be considered relevant if compared with our previous evaluations in other anatomical sites [17, 18].

Looking the SD for the cranio-caudal, anterio-posterior, and latero-lateral diameters of the Elective CTVs, the greatest variation was observed in the cranio-caudal diameter (2.7 and 2.5 in post-operative and pre-operative case, respectively) (Tables 2), especially in the caudal direction (Figure 2). These discrepancies could be related to the fact that for head of pancreas lesions, the inferior limit of RT standard field is often considered at the level of the second or third lumbar vertebra (L2-L3) [24, 25]. This, in some centres, may have led to a misunderstanding compared to than suggested by Goodman KA et al. [12] in adjuvant setting (bottom of the third lumbar vertebra) and by Caravatta L et al. [14] for neoadjuvant/exclusive setting (caudal margin matches with the inferior mesenteric artery origin) for tumors sited at the uncinate process, as in both proposed cases.

The relatively low variability of the Elective CTVs, in both cases, was confirmed by the mDSI of 0.68 in both cases. In addition, the mDSI was increased up to 0.71 (post-operative RT) and 0.72 (pre-operative RT) when the observers with a worse agreement with the reference centre have been excluded. To the best of our knowledge there are no experiences evaluating DSI value for the inter-observer variability in the volumes delineation of abdominal RT. Furthermore, although values of DSI up to 0.85 have been reported for other anatomical sites (prostate, lung, and breast, i.e.) [20, 27] it is believed that 0.68 obtained in our study can be considered a good value, taking in account that it is referred to the delineation of lymph node areas rather than to a well-defining organ, such as the prostate.

The significant variability in Boost CTV delineation in the post-operative case (Case 1, Table 3) might be related to a more difficult identification of the reference structures in the post-surgery imaging.

Otherwise, regarding pre-operative RT (Case 2), the lack of recognized guidelines about the GTV margin to define the CTV could be identified as the main cause of the increased variability of the Boost CTV compared to the Elective CTV (Table 3). Indeed, studies on pathologic reports suggest margins from 10 to 30 mm around the GTV [5, 28]. This might have led to the larger variability in comparison to the study by Yamazaki H et al., evaluating the inter-observer variance in GTV delineating in patients with unresectable pancreatic cancer. The mean GTV of the pancreatic head cancer was 34.8 cc (SD, 30.4 cc; median, 31.8 cc; range, 13.5-122 cc) [29], however in Yamazaki’s study the radiation field was fitted to GTV (CTV = GTV) without ENI.

Furthermore, looking to the qualitative analysis (Table 1), some critical issues that may have an impact on the variability in the definition of CTVs in routine clinical practice can be detected. The administration of intravenous contrast during planning CT scan as well as the merging with the staging imaging set seems not to be a primary requisite for the identifications of the reference structures. In fact, only 6 centres (33.3%) declared to routinely use intravenous contrast-enhanced planning CT scan and performed co-registration of staging imaging with the planning CT scan. A study where contrast-enhanced planning CT scan is used could be rescheduled to assess a probable reduction of inter-observer variability.

On the other hand, programs of collaboration with radiologists and/or nuclear physician are not routinely applied (Table 1). In fact, 15 radiation oncologists (83.3%) stated to require the collaboration of radiologists and/or nuclear physician for CTVs delineation only for cases of more difficult interpretation. Finally, 8 centres (44.4%) reported that they never or almost never require MRI or CT-PET for CTVs contouring in daily clinical practice.

The use of a CT-simulation without use of intravenous contrast could be represent one limitation of this study, but actually it was our precise choice aimed to give freedom in set-up defining according to own protocols and to get as much as possible closer to routinely clinical practice, given that in most Italian and European centres, the CT-simulation is performed without intravenous contrast. In order to evaluate the possibility of improving the results recorded in this analysis, we plan a further study in which the interobserver variability will be assessed on the basis of a contrast-enhanced CT and / or MRI image fusion with CT-simulation images.

Further limitation of our analysis is that it does not provide information about PTV margins, because the main aim was to evaluate the interobserver variability only in terms of CTV. Since significant changes may be also affected by the organ motion and more generally by the margins from CTV to PTV, further analysis are needed to evaluate this important potential source of variability in the definition of the target, as well as to determine the potential dosimetric impact of an incorrect definition of the target, both with standard (3D-CRT) and advanced (IMRT, VMAT) techniques.

## Conclusions

The obtained values of CV and mDSI for Elective CTVs showed an acceptable agreement, in both post-operative and pre-operative case. Based on these results we can conclude that the availability of national reference criteria has produced good results. Additional strategies to further increase the agreement in Elective CTV and reduce variability in Boost CTV delineation may be sought by implementing the routinely use of intravenous contrast-enhanced planning CT scan and multi-modality imaging co-registration techniques and by promoting collaborative multidisciplinary programs involving radiologists and/or nuclear physician.

## Authors’ information

Alessio G Morganti and Domenico Genovesi share senior authorship.

## References

[1] Morganti AG, Massaccesi M, La Torre G, Caravatta L, Piscopo A, Tambaro R, Sofo L, Sallustio G, Ingrosso M, Macchia G, Deodato F, Picardi V, Ippolito E, Cellini N, Valentini V (2010). A systematic review of resectability and survival after concurrent chemoradiation in primarily unresectable pancreatic cancer. Ann Surg Oncol.

[2] Goodman KA, Hajj C (2013). Role of radiation therapy in the management of pancreatic cancer. J Surg Oncol.

[3] Asiyanbola B, Gleisner A, Herman JM, Choti MA, Wolfgang CL, Swartz M, Edil BH, Schulick RD, Cameron JL, Pawlik TM (2009). Determining pattern of recurrence following pancreaticoduodenectomy and adjuvant 5-flurouracil-based chemoradiation therapy: effect of number of metastatic lymph nodes and lymph node ratio. J Gastrointest Surg.

[4] Yoshida T, Matsumoto T, Sasaki A, Shibata K, Aramaki M, Kitano S (2004). Outcome of paraaortic node-positive pancreatic head and bile duct adenocarcinoma. Am J Surg.

[5] Hishinuma S, Ogata Y, Tomikawa M, Ozawa I, Hirabayashi K, Igarashi S (2006). Patterns of recurrence after curative resection of pancreatic cancer, based on autopsy findings. J Gastrointest Surg.

[6] Crane CH, Antolak JA, Rosen II, Forster KM, Evans DB, Janjan NA, Charnsangavej C, Pisters PW, Lenzi R, Papagikos MA, Wolff RA (2001). Phase I study of concomitant gemcitabine and IMRT for patients with unresectable adenocarcinoma of the pancreatic head. Int J Gastrointest Cancer.

[7] Yovino S, Poppe M, Jabbour S, David V, Garofalo M, Pandya N, Alexander R, Hanna N, Regine WF (2011). Intensity-modulated radiation therapy significantly improves acute gastrointestinal toxicity in pancreatic and ampullary cancers. Int J Radiat Oncol Biol Phys.

[8] Caravatta L, Macchia G, Deodato F, Felicetti M, Cellini F, Ciabattoni A, Buwenge M, Picardi V, Cilla S, Scapati A, Valentini V, Morganti AG (2013). Intensity-Modulated radiotherapy in the treatment of pancreatic adenocarcinoma: a review. EMJ Oncol.

[9] NCCN (2013). Clinical Practice Guidelines in Oncology, Pancreatic Adenocarcinoma.

[10] Fiorino C, Reni M, Bolognesi A, Cattaneo GM, Calandrino R (1998). Intra- and inter-observer variability in contouring prostate and seminal vesicles: implications for conformal treatment planning. Radiother Oncol.

[11] Brunner TB, Merkel S, Grabenbauer GG, Meyer T, Baum U, Papadopoulos T, Sauer R, Hohenberger W (2005). Definition of elective lymphatic target volume in ductal carcinoma of the pancreatic head based on histopathologic analysis. Int J Radiat Oncol Biol Phys.

[12] Goodman KA, Regine WF, Dawson LA, Ben-Josef E, Haustermans K, Bosch WR, Turian J, Abrams RA (2012). Radiation Therapy Oncology Group consensus panel guidelines for the delineation of the clinical target volume in the postoperative treatment of pancreatic head cancer. Int J Radiat Oncol Biol Phys.

[13] Sun W, Leong CN, Zhang Z, Lu JJ (2010). Proposing the lymphatic target volume for elective radiation therapy for pancreatic cancer: a pooled analysis of clinical evidence. Radiat Oncol.

[14] Caravatta L, Sallustio G, Pacelli F, Padula GD, Deodato F, Macchia G, Massaccesi M, Picardi V, Cilla S, Marinelli A, Cellini N, Valentini V, Morganti AG (2012). Clinical target volume delineation including elective nodal irradiation in preoperative and definitive radiotherapy of pancreatic cancer. Radiat Oncol.

[15] Associazione Italiana Radioterapia Oncologica (AIRO) - Gruppo di Studio per i Tumori Gastrointestinali (2012). Tumori Gastrointestinali - Indicazioni e Criteri Guida. Cap.

[16] Japan Pancreas Society: **Classification of pancreatic carcinoma.** Tokyo: Kanehara; 2003. 2nd English

[17] Genovesi D, Ausili-Cèfaro G, Vinciguerra A, Augurio A, Di Tommaso M, Marchese R, Ricardi U, Filippi AR, Girinsky T, Di Biagio K, Belfiglio M, Barbieri E, Valentini V (2011). Interobserver variability of clinical target volume delineation in supra-diaphragmatic Hodgkin’s disease: a multi-institutional experience. Strahlenther Onkol.

[18] Genovesi D, Ausili-Cèfaro G, Trignani M, Vinciguerra A, Augurio A, Di Tommaso M, Perrotti F, De Paoli A, Olmi P, Valentini V, Di Nicola M: **Interobserver variability of clinical target volume delineation in soft-tissue sarcomas.** *Cancer/Radiothérapie* Available online 17 January 2014. In press 10.1016/j.canrad.2013.11.011 24440683

[19] Dice LR (1945). Measures of the amount of ecologic association between species. Ecology.

[20] Fotina I, Lütgendorf-Caucig C, Stock M, Pötter R, Georg D (2012). Critical discussion of evaluation parameters for inter-observer variability in target definition for radiation therapy. Strahlenther Onkol.

[21] Fokas E, Eccles C, Patel N, Chu KY, Warren S, McKenna WG, Brunner TB (2013). A treatment planning comparison of four target volume contouring guidelines for locally advanced pancreatic cancer radiotherapy. Radiother Oncol.

[22] Abrams RA, Winter KA, Regine WF, Safran H, Hoffman JP, Lustig R, Konski AA, Benson AB, Macdonald JS, Rich TA, Willett CG (2012). Failure to adhere to protocol specified radiation therapy guidelines was associated with decreased survival in RTOG 9704--a phase III trial of adjuvant chemotherapy and chemoradiotherapy for patients with resected adenocarcinoma of the pancreas. Int J Radiat Oncol Biol Phys.

[23] Willett CG, Moughan J, O’Meara E, Galvin JM, Crane CH, Winter K, Manfredi D, Rich TA, Rabinovitch R, Lustig R, Machtay M, Curran WJ (2012). Compliance with therapeutic guidelines in Radiation Therapy Oncology Group prospective gastrointestinal clinical trials. Radiother Oncol.

[24] Metha VK, Lu JJ, Brady LW (2008). Radiation Oncology: An Evidence-Based Approach.

[25] Halperin EC, Perez CA, Brady LW (2008). Principles and Practice of Radiation Oncology.

[26] Brunner TB, Baum U, Grabenbauer GG, Sauer R, Lambrecht U (2006). Large topographic variability of upper abdominal lymphatics and the consequences for radiation treatment planning. Radiother Oncol.

[27] Batumalai V, Koh ES, Delaney GP, Holloway LC, Jameson MG, Papadatos G, Lonergan DM (2011). Interobserver variability in clinical target volume delineation in tangential breast irradiation: a comparison between radiation oncologists and radiation therapists. Clin Oncol (R Coll Radiol).

[28] Crippa S, Partelli S, Falconi M (2010). Extent of surgical resections for intraductal papillary mucinous neoplasms. World J Gastrointest Surg.

[29] Yamazaki H, Nishiyama K, Tanaka E, Koiwai K, Shikama N, Ito Y, Arahira S, Tamamoto T, Shibata T, Tamaki Y, Kodaira T, Oguchi M (2007). Dummy run for a phase II multi-institute trial of chemoradiotherapy for unresectable pancreatic cancer: inter-observer variance in contour delineation. Anticancer Res.

